# Women with large (≥3 cm) and locally advanced breast cancers (T3, 4, N1, 2, M0) receiving neoadjuvant chemotherapy (NAC: cyclophosphamide, doxorubicin, docetaxel): addition of capecitabine improves 4-year disease-free survival

**DOI:** 10.1186/2193-1801-4-9

**Published:** 2015-01-13

**Authors:** Jennifer Eremin, Ged Cowley, Leslie G Walker, Elisabeth Murray, Monika Stovickova, Oleg Eremin

**Affiliations:** 9 Research & Development Department, Lincoln County Hospital, Greetwell Road, Lincoln, LN2 5QY UK; 10 Lincoln Breast Unit, Lincoln County Hospital, Greetwell Road, Lincoln, UK; 11 Department of Pathology, PathLinks, Lincoln County Hospital, Greetwell Road, Lincoln, UK; 12 Department of Oncology, Lincoln County Hospital, Greetwell Road, Lincoln, UK; 13 Department of Radiology, Lincoln County Hospital, Greetwell Road, Lincoln, UK; 14 Medical Research Centre, University of Hull, Hull, UK; 15 Division of Surgery, The University of Nottingham, Queen’s Medical Centre, Derby Road, Nottingham, UK

**Keywords:** Breast cancer, Neoadjuvant chemotherapy, Response, Survival

## Abstract

**Purpose:**

To determine whether capecitabine (X), combined with docetaxel (T) following doxorubicin (A) and cyclophosphamide (C), enhanced the pathological complete response (pCR) in the breast and axillary lymph nodes (ALNs) of women with large or locally advanced breast cancers (LLABCs) improving outcome, and the effect on quality of life (QoL).

**Patients and methods:**

117 women were enrolled, 112 randomised to 2 cycles of AC (60 mg/m^2^, 600 mg/m^2^) given 3 weekly. Tumour responses were assessed by magnetic resonance mammography. Responders (n = 77) received 2 further cycles of AC and were randomised to 4 cycles of T (100 mg/m^2^) (Group A) or T (75 mg/m^2^) and X (2000 mg/m^2^/day), day one to 14 of each 3 weekly cycle (Group B). Non-responders (n = 35) were randomised to 6 cycles of T (Group C) or T + X (Group D). QoL questionnaires were completed at each chemotherapy visit. Pathological responses were evaluated using established criteria.

**Results:**

The groups were comparable in patient and tumour characteristics (79.5% T2, 85.7% ductal, 73.2% ER +ve, 22.3% HER2 +ve, 42% involved ALNs). Overall breast pCR was 27.1%, Groups A + C versus B + D (p = 0.446). ALN +ve pCR was 41.9%, Groups A + C versus B + D (p = 0.231). 4-year disease-free survival (DFS) was significantly improved with X (p = 0.016) but not overall survival (p = 0.056). Triple -ve and HER2 +ve tumours, and persistent ALN disease were risk factors for metastases. X increased severe nail changes (p = 0.0002) and hand-foot syndrome (p = 0.014) without affecting QoL.

**Conclusion:**

NAC-X did not increase breast and ALN pCR but improved 4-year DFS, without detriment to QoL.

## Introduction

Neoadjuvant chemotherapy (NAC) is used to treat patients with large or locally advanced breast cancers (LLABCs), to downstage the disease and perform breast conserving surgery (Kaufmann et al. 2012; Schott and Hayes 2012). NAC is not detrimental to patient survival and is comparable in efficacy with adjuvant chemotherapy (Schott and Hayes 2012; Mieog et al. 2007).

The addition of taxanes and trastuzumab to NAC combinations has improved the pathological complete response (pCR) in the breast and axillary lymph nodes (ALNs) (Kaufmann et al. 2012; Smith et al. 2002; Bear et al. 2006; Semiglazov et al. 2011). The addition of capecitabine has also been studied; shown to be effective in combination with taxanes in women with metastatic disease (O'Shaughnessy 2002; Gluck et al. 2013).

Our study commenced in November 2008, at which time phase 2 studies had shown increased pCR in the breast with capecitabine combinations (Lebowitz et al. 2004; Villman et al. 2007).

A pCR in the breast and axilla is a surrogate marker of long-term disease-free survival (DFS) and overall survival (OS) (Kaufmann et al. 2012; Penault-Llorca et al. 2008; von Minckwitz et al. 2012). Patients with a breast pCR but residual cancer in the ALNs, however, have a poor prognosis (Mazouni et al. 2007; Marchio and Sapino 2011). A pCR in the breast occurs in 17% to 30% of patients using various anthracycline-taxane combinations and histopathological criteria for assessing pCR (Smith et al. 2002; Bear et al. 2006; von Minckwitz et al. 2010; Walker et al. 2011). The use of trastuzumab with human epidermal growth factor receptor 2 positive (HER2 +ve) cancers has further increased the pCR responses (Kaufmann et al. 2012; Semiglazov et al. 2011).

Two studies have reported an improved pCR with capecitabine (Lee et al. 2008; Steger et al. 2010). Three studies failed to find an enhanced pCR (von Minckwitz et al. 2010; Bear et al. 2012; Ohno et al. 2013). A recent meta-analysis of anthracycline-taxane combinations did not demonstrate any significant increase of pCR. Adding capecitabine to NAC regimens is unlikely to improve outcomes in breast cancer in patients without distant metastases (Li et al. 2013). However, LLABC should be regarded as a systemic disease with local manifestations.

Spread to ALNs carries a poor prognosis (Carter et al. 1989; Recht and Houlihan 1995). Even in the absence of ALN invasion, 20% of women with early breast cancer die from metastases (Fisher et al. 2010). Patients with LLABCs are at risk of early tumour recurrence following NAC combinations of anthracycline, taxanes and cyclophosphamide. The Lincoln study showed a 74% 5-year DFS, whilst the NSABP-27 study a 62% 8-year DFS (Bear et al. 2006; Walker et al. 2011). Hence, many women without evidence of distant disease harbour occult micro-metastases.

New NAC combinations are being evaluated to determine their effect on pCR and assess morbidity and survival benefit (Kaufmann et al. 2012; Schott and Hayes 2012; Colleoni and Goldhirsch 2014).

The aims of our study were to evaluate the effect of capecitabine in anthracycline-taxane NAC combinations, on pCR in the breast and ALNs, quality of life (QoL), DFS and OS.

## Patients and methods

### Patients and eligibility

Women (18–75 years) with LLABCs (≥3 cm,T3,4, N1,2, M0) with a WHO performance of ≤2, satisfactory haematological, renal, hepatic and cardiac function (absolute neutrophil count ≥1500/μl, platelet count ≥100,000/μl; total bilirubin <20 μmol/L, alkaline phosphatase, transaminases <2 x upper limit of normal; serum creatinine <2.0 mg/dL; left ventricular ejection fraction ≥50% by echocardiography), were invited to participate.

Exclusion criteria were pregnancy, lactation, previous malignancy other than basal carcinoma of skin, insulin dependent diabetes, inability to give informed consent or to complete QoL questionnaires.

The study was approved by the Regional Research Ethics Committee on 24/01/2008 (Rec No: 07/H0406/260). Participants signed an Informed Consent.

### Study design

Diagnosis was established by clinical examination, mammography, ultrasonography and histological assessment of needle biopsies.

Patients underwent a chest radiograph and liver ultrasound scan (USS) or computerised tomography of thorax and abdomen, bone scintigraphy, electrocardiography, echocardiography and a magnetic resonance mammogram (MRM) prior to commencing treatment.

QoL was assessed using validated questionnaires (Walker et al. 2011). Prior to each cycle, and after NAC, the Hospital Anxiety and Depression Scale (HADS), Mood Rating Scale (MRS) and Treatment Side-Effects Questionnaires were completed (Zigmond and Snaith 1983; Sharp et al. 2010; Walker et al. 1998). Before cycles one, 5 and after completion of NAC, a Patient Satisfaction Questionnaire and the Functional Assessment of Cancer Therapy (Breast) (FACT-B) with Taxane (T) modules were completed (Brady et al. 1997). The primary QoL outcome was the Trial Outcome Index (TOI) of FACT-B at follow-up (Brady et al. 1997).

Patient enrolment, randomisation, treatment and assessment are outlined in the CONSORT diagram.

The study registration number is ISRCTN 00407556.

Randomisation was carried out according to the MRM response after 2 courses of NAC, using permuted blocks. Treatment allocation was determined using sealed sequential envelopes from Responder and Non-responder containers.

### NAC regimen

Patients received 3 weekly IV doxorubicin (A: 60 mg/m^2^) and cyclophosphamide (C: 600 mg/m^2^) for two cycles.

Responders were randomised into Group A or B. Both groups received 2 further 3 weekly IV AC followed by 4 cycles of IV docetaxel (T) 100 mg/m^2^ every 3 weeks (Group A) or 4 cycles of IV T 75 mg/m^2^ and O capecitabine (X) 2000 mg/m^2^/day for 14 days every 3 weeks (Group B).

Non-responders were randomised into Group C and D. Group C received 6 cycles of IV T 100 mg/m^2^ every 3 weeks. Group D received 6 cycles of IV T 75 mg/m^2^ and O X 2000 mg/m^2^/day for 14 days every 3 weeks.

Ondansetron and dexamethasone were prescribed during and after each cycle.

Patients received lenograstim 263 μgm SC, days 2–6 after each cycle from cycle 3 onwards. If febrile neutropenia occurred with cycle one, lenograstim was given from cycle 2 onwards.

### Surgery and radiotherapy

Prior to NAC, a radio-opaque marker was inserted into the breast tumour, enabling needle localisation of the tumour during breast conservation.

Wide local excision or mastectomy, according to surgical advice (or patient preference), and either ALN sampling (at least 4 nodes) or axillary clearance (preNAC involved ALNs) was performed approximately 4 weeks following chemotherapy.

Following breast conservation, patients received radiotherapy to the breast. If node sampling established involvement, the axilla and supraclavicular region were irradiated. Following mastectomy, chest wall irradiation was given if the patient was deemed at risk of local recurrence.

Two patients presenting with T4 tumours received radiotherapy to the breast and draining lymph nodes prior to surgery.

Patients whose tumours were oestrogen receptor positive (ER +ve) received tamoxifen 20 mg O daily if premenopausal, anastrozole 1 mg O daily/letrozole 2.5 mg O daily if postmenopausal, for 5 years. Patients with HER2 +ve tumours underwent a 3 weekly x 18 course of IV trastuzumab (8 mg/kg for the first two cycles, 6 mg/kg in subsequent cycles).

### Assessment of response

Clinical: Caliper measurements were carried out at each cycle and prior to surgery. Ultrasonographic measurements were performed after cycles 4, 6 and 8; mammograms (MMGs) after cycles 4 and 8. RECIST criteria were used to evaluate responses (Eisenhauer et al. 2009).

Pathological responses were evaluated in the surgical specimens. These were graded 1 to 5 in the breast; pCR had no invasive disease (DCIS permitted), fibroelastic scar or hyaline amorphous area. ALNs were graded 1 to 3; pCR had no tumour cells, with or without areas of fibrosis.

### Statistical analysis

Data were analysed using SPSS v22. Pearson’s product–moment correlations were used to explore relationships between selected variables. Between-group comparisons were carried out at baseline using Analysis of Variance (ANOVA) and Chi-square as appropriate, and at subsequent time-points using Analysis of Co-variance (ANCOVA) with baseline values as covariates (Vickers and Altman 2001).

Survival data were evaluated using Kaplan-Meier curves and significance established using the Log Rank test. The small number of deaths and pCRs precluded the use of Cox proportional hazards and other multivariate methods to identify independent prognostic factors. Analysis was by intention-to-treat; alpha was set at <0.05 (two-tailed) (Peduzzi et al. 1995).

## Results

### Treatment groups

From November 2008 to October 2011, 117 women with LLABCs were enrolled and 112 randomised following MRM assessment after 2 cycles of AC.

Three patients had conditions not initially detected requiring study withdrawal. One patient withdrew due to a severe reaction to AC and one withdrew consent after one course of chemotherapy.

Of the 112 patients randomised, 77 (69%) were responders and were randomised to Group A (38) or B (39); 35 (31%) were non-responders and were randomised to Group C (19) or D (16) (CONSORT diagram).

Following randomisation, two patients (Group A, B) withdrew consent. Chemotherapy was discontinued due to progressive disease in 6 patients: 3 after 4 cycles and 3 after 6 cycles. Ninety seven (88.2%) patients completed 7 cycles and 92 (83.6%) 8 cycles.

One hundred and ten patients underwent surgery and subsequent radiotherapy, as appropriate.

### Patient and tumour characteristics

The ACT and ACTX therapeutic groups were comparable in terms of age, menopausal status and tumour characteristics – TNM classification, tumour size, type and grade, nodal involvement, ER and HER2 status (Table 1).

**Table 1:** **Patient and tumour characteristics of randomised patients**

	GROUPS A + C (n = 57)	STATISTICAL SIGNIFICANCE*		GROUPS B + D (n = 55)
		***χ*** ^2^	p-value	
Age in years, mean (range)	53.9 (33 – 69)	-	-	53.0 (35 – 69)
Menopausal status, n (%)				
PRE-PERI	24 (42.1)	0.572	0.449	29 (52.7)
POST	33 (57.9)			26 (47.3)
TNM Classification, n (%)				
T0-T1	2 (3.5)			1 (1.8)
T2	43 (75.4)			46 (83.6)
T3	5 (8.8)	4.881	0.300	5 (9.1)
T4	7 (12.3)			3 (5.5)
N0	32 (56.1)			29 (52.7)
N1-2	25 (43.9)			26 (47.3)
Tumour size in cm (caliper), mean (range)	4.3 (2 – 14)	-	-	4.2 (1.9 – 14)
Pre NAC nodal involvement, confirmed by biopsy, n (%)	22 (39.3)	-	-	23 (42.6)
Tumour type, n (%)				
Ductal invasive	49 (86.0)	0.410	0.815	47 (85.5)
Lobular invasive (and others)	8 (14.0)			8 (14.5)
Tumour grade, n (%)				
Grades 1 and 2	38 (66.7)	2.495	0.287	28 (51.0)
Grade 3	19 (33.3)			27 (49.0)
ER status, n (%)^a^				
Positive (Allred >3)	45 (78.9)	1.732	0.421	37 (67.3)
Negative (Allred < 3)	12 (21.1)			12 (32.7)
HER2 status, n (%)^b^				
Positive (FISH)	11 (19.3)	0.212	0.450	14 (25.9)
Negative	46 (80.7)			41 (74.1)

^a^ER : Oestrogen receptor. ^b^HER2: Human epidermal growth factor receptor 2. Allred : Scoring system for measuring expression of oestrogen receptors in tissue sections. FISH : Fluorescence in-situ hybridisation.

*Various patient and tumour characteristics were not significantly different between Groups (A + C) and (B + D) (p>0.05, Pearson Chi-Square Test).

The mean age was 53 years (range 33 to 69). Fifty-three were pre-menopausal/ peri-menopausal, 59 post-menopausal. The majority of tumours were T2 (79.5%), ductal invasive (85.7%), ER +ve (73.2%) and HER2 -ve (77.7%), and 45 (42.2%) patients had involved ALNs (Table 1). Seventy one tumours were ER +ve HER2 -ve (luminal A), 15 ER +ve HER2 +ve (luminal B), 9 ER -ve PR -ve HER2 +ve (HER2 overexpressing) and 15 ER -ve PR -ve HER2 -ve (basal-like).

### Clinical response

Of the 110 patients who were assessed (one had involved ALNs only), 51 in Groups A + C and 49 in B + D achieved a CR or PR. The CR + PR in ACT was 91.9% and 92.5% in ACTX (*χ* ^2^ = 0.715, p = 0.398).

### Imaging response

Ninety-five patients had lesions evaluable by ultrasonography, whilst only 74 by digital mammography. Failure to detect by mammography occurred in younger women and/or in those with dense breasts. In 5 patients the imaging assessment was incomplete.

There was poor concordance between the responses detected on ultrasonography and mammography. USSs and MMGs were unreliable assessors of pathological responses in the breast (data not shown).

### Pathological response

Table 2 documents pathological responses in the breast; 107 breast specimens were available for assessment. The overall pCR was 29 (27.1%); exclusion of DCIS reduced this to 20 (18.7%). The pCR with ACT was 22.2% and with ACTX 32.1% (*χ* ^2^ = 3.717, p = 0.446). PCR occurred in 17% of luminal A, 47% of luminal B, 33% of HER2 overexpressing, and 50% of basal-like tumours. Triple negative and HER2 +ve tumours had the highest pCR, whilst luminal A the lowest.

**Table 2:** **Pathological response in breast [n (%)] following NAC**

	PATHOLOGICAL RESPONSE GRADE [n(%)] ^b^ *			
GROUPS (n = 107) ^a^	5(PCR)	4(MRD)	3(PR)	2/1(PoR/NR)
A	11 (30.6)	10 (27.8)	10 (27.8)	5 (13.9)
(n = 36)				
B	15 (40.5)	10 (27)	9 (24.3)	3 (8.1)
(n = 37)				
C	1 (5.6)	1 (5.6)	6 (27.8)	10 (16.1)
(n = 18)				
D	2 (12.5)	2 (12.5)	5 (31.3)	7 (43.8)
(n = 16)				
(A + C)	12 (22.2)	11 (20.4)	15 (27.8)	16 (29.6)
(n = 54)				
(B + D)	17 (32.1)	12 (22.6)	14 (26.4)	10 (18.8)
(n = 53)				

^a^Two patients received preoperative radiotherapy and one had no demonstrable cancer in the breast (involved ALN on presentation).

^b^PCR : complete response, no residual invasive tumour cells in specimen, DCIS accepted (grade 5); MRD : minimal residual disease, >90% loss of tumour cells, (grade 4); PR : partial response, moderate reduction in tumour cell burden, between 30%-90% reduction in tumour cells (grade 3); PoR : poor response, minimal loss (<30%) of tumour cells, (grade 2); NR : no response/no change in overall cellularity, (grade 1).

*Pathological responses were not significantly different between the Groups (p>0.05); A + C versus (v) B + D: *χ* ^2^ = 3.717, p = 0.446 (Pearson Chi-Square Test).

Table 3 documents pathological response in ALNs;108 specimens were available for assessment. On presentation 38.6% of ALNs in Groups A + C and 41.8% in B + D contained metastases. The overall pCR in the ALNs was 16.7%, and 41.9% in the pre NAC involved ALNs. There was no significant increase in pCR in either subset with ACTX (*χ* ^2^ = 2.743, p = 0.098; *χ* ^2^ = 1.433, p = 0.231).

**Table 3:** **Pathological response in excised axillary nodes following NAC**

GROUPS (n = 110) ^a^	PATHOLOGICAL RESPONSE GRADE [n (%)] ^b^ *			PRE NAC NORMAL NODES (n = 67)	PRE NAC TUMOUR INVOLVED ALNs (n = 45)	POST NAC PCR in ALNs (n = 43) ^a^
	3 (PCR)	2 (PR)	1(PoR)			
A^a^	6 (16.7%)	4 (11.1%)	4 (11.1%)	21 (55.3%)	17 (44.7%)	6 (35.3%)
n = 37						
B^a^	8 (21.1%)	4 (10.5%)	3 (7.9%)	23 (60.5%)	15 (39.5%)	8 (53.3%)
n = 38						
C	0 (0%)	2 (11.1%)	2 (11.1%)	14 (77.8%)	5 (26.3%)	0 (0%)
n = 19						
D	4 (25.0%)	2 (12.5%)	1 (6.25%)	9 (56.3%)	8 (50%)	4 (50%)
n = 16						
A + C	6 (11.1%)	6 (11.1%)	6 (11.1%)	35 (61.4%)	22 (38.6%)	6 (30%) (n = 20)
n = 56						
B + D	12 (22.2%)	6 (11.1%)	4 (7.4%)	32 (58.2%)	23 (41.8%)	12 (52.2%) (n = 23)
n = 54						

^a^Two patients had pre-operative radiotherapy and were excluded from the post NAC analysis, one in Group A and one in Group B.

^b^PCR : complete response, all metastatic disease replaced by fibrosis (grade 3); PR : partial response in metastatic disease and evidence of fibrotic replacement of malignant cells (grade 2); PoR : poor response, metastasis with no evidence of fibrosis (grade 1); normal nodes: no evidence of metastatic disease or fibrosis in lymph nodes.

*Pathological responses were not significantly different between the Groups (p>0.05); A + C v B + D (all ALNs): *χ* ^**2**^ = 2.743, p = 0.098; A + C v B + D (pre NAC +ve ALNs): *χ* ^2^ = 1.433, p = 0.231 (Pearson Chi-Square Test).

There was a 24.4% (n = 11) concurrent pCR in both the breast and ALNs, a threefold increase with ACTX, suggesting a possible benefit with capecitabine.

There were significant correlations between the pathological responses in the breast and tumour grade (r_xy_ = 0.319, p = 0.001) and HER2 status (r_xy_ = - 0.213, p = 0.029).

### NAC and toxicity

There were no deaths in the study; 13 patients experienced Serious Adverse Events and one a Suspected Unexpected Serious Adverse Reaction. The anticipated side-effects of chemotherapy (alopecia, nausea, vomiting, excess lacrimation, blood-stained nasal discharge and fatigue) were commonly seen, treated as appropriate, and were well tolerated by most patients.

Twenty-four patients had 28 episodes of febrile neutropenia requiring hospitalisation for one to 6 days; 24 occurred during the first two cycles prior to commencing lenograstim. Lenograstim substantially reduced the incidence of febrile neutropenia, preventing delays in treatment and minimising the need for dose reduction.

Grade 3/4 toxicities following completion of 8 cycles of NAC are shown in Table 4.

**Table 4:** **Grade 3 and 4 toxicities [n (%)] associated with NAC, evaluated at cycle 8** ^a^

GROUPS (n = 92)	TOXICITY				
	NAILS*	HAND-FOOT*	PARESTHESIA	MYALGIA*	FATIGUE
A	6(20.0%)	1 (3.3%)	2 (6.6%)	7 (23.3%)	16 (53.3%)
(n = 30)					
B	16 (47.1%)	7 (23.3%)	1 (2.9%)	1 (2.9%)	21 (61.7%)
(n = 34)					
C	1 (6.3%)	0 (0%)	2 (12.5%)	1 (6.3%)	9 (56.3%)
(n = 16)					
D	8 (66.7%)	1 (8.3%)	2 (16.6%)	0 (0%)	9 (75.0%)
(n = 12)					
(A + C)	7 (15.2%)	1 (2.2%)	4 (8.7%)	8 (17.4%)	25 (54.3%)
(n = 46)					
(B + D)	24 (52.2%)	8 (17.4%)	3 (6.5%)	1 (2.2%)	30 (65.2%)
(n = 46)					

^a^Serious Adverse Events (SAEs) : n = 13; Suspected Unexpected Serious Adverse Reactions (SUSARs) : n = 1; Deaths : n = 0. NS : Not Significant.

*Statistically significant (Pearson Chi-Square Test). *Nail changes : A v B (*χ* ^2^ = 5.173, p = 0.023); C v D (*χ* ^2^ = 11.476, p = 0.007); A + C v B + D (*χ* ^2^ = 14.060, p = 0.0002). *Hand-foot syndrome : A v B (*χ* ^2^ = 4.338, p = 0.037); C v D (NS); A + C v B + D (*χ* ^2^ = 6.035, p = 0.014). Paresthesia : A v B (NS); C v D(NS); A + C v C + D (NS). *Myalgia : A v B (*χ* ^2^ = 9.572, p = 0.023); A + C v B + D (*χ* ^2^ = 6.035, p = 0.014). Fatigue : A v B (NS); C v D (NS); A + C v B + D (NS).

Pronounced fatigue was very common (>50%) but was comparable between groups (*χ* ^2^ = 1.813, p = 0.612). Grade 3/4 myalgia occurred in <25% and was seen predominantly in patients receiving T (100 mg/m^2^): Groups A + C versus B + D (*χ* ^2^ = 6.035, p = 0.014). Severe paresthesia occurred in <20% and was comparable between groups (*χ* ^2^ = 3.037, p = 0.386).

Severe hand-foot syndrome was more common, with the use of X: Groups A + C versus B + D (*χ* ^2^ = 6.035, p = 0.014).

Severe nail changes were very common, predominantly in patients receiving X: Groups A + C versus B + D (*χ* ^2^ = 14.060, p = 0.0002).

### QoL and NAC

The primary QoL outcome was the TOI of FACT-B. Groups A + C and B + D did not differ at baseline (F = 0.407, df = 1, p = 0.482), before cycle 5 (F = 0.921, df = 2, p = 0.339) or at follow-up (F = 0.022, df = 2, p = 0.882). In addition, there were no significant differences at any of these three time-points for any of the FACT subscales (Physical, Social, Family, Emotional and Functional well-being, Breast Cancer and Taxane).

Similarly, Groups A + C and B + D did not differ at any time-point on any of the secondary outcome measures.

TNM tumour (T) correlated positively with anxiety (r_xy_ = 0.296, p = 0.001) and depression (r_xy_ = 0.291, p = 0.002), and negatively with happiness (r_xy_ = -0.185, p = 0.049), clear headedness (r_xy_ = -0.249, p = 0.008) and relaxation (r_xy_ = -0.236, p = 0.011) (Table 5).

**Table 5:** **Significant (2-tailed) correlations between various parameters in women with LLABCs undergoing NAC**

	PSYCHOLOGICAL PARAMETERS ^a^	STATISTICAL SIGNIFICANCE*	
		r _xy_	p-value
**TNM TUMOUR (T) CLASSIFICATION**	HADS (ANXIETY)	0.296	0.001
	HADS (DEPRESSION)	0.291	0.002
	MRS (HAPPINESS)	-0.185	0.049
	MRS (CLEAR HEADEDNESS)	-0.249	0.008
	MRS (RELAXATION)	-0.236	0.011
**CLINICAL RESPONSE IN BREAST TO NAC**	HADS (DEPRESSION)	0.218	0.020
	MRS (HAPPINESS)	-0.217	0.021
	MRS (CLEAR HEADEDNESS)	-0.217	0.021
**PATHOLOGICAL RESPONSE IN BREAST TO NAC**	MRS (CLEAR HEADEDNESS)	0.263	0.010
	MRS (CONFIDENCE)	0.266	0.009

^a^HADS : Hospital anxiety and depression scales; MRS : Mood rating scales.

*r_xy_ : Pearson Correlation (Univariate Analysis of Variance).

Clinical response correlated positively with depression (r_xy_ = 0.218, p = 0.020), and negatively with clear headedness (r_xy_ = -0.217, p = 0.021) and happiness (r_xy_ = -0.217, p = 0.021).

Pathological response correlated positively with clear headedness (r_xy_ = 0.263, p = 0.010) and confidence (r_xy_ = 0.266, p = 0.009), 3 weeks post NAC and before surgery.

### Metastatic disease and survival

The first patient was randomised in December 2008 and the last in October 2011. Following randomisation, 110 patients were followed-up from 30 to 64 months – 56 patients in Groups A + C and 54 in Groups B + D.

Twelve patients (21.2%) who received ACT and 3 (5.6%) ACTX have developed metastases (liver, lung, bone, brain): 8 (14.3%) who received ACT and 2 (3.7%) ACTX have died. Patients with basal-like (triple -ve) and HER2 overexpressing tumours were at high risk of developing metastases.

No patient who had a pCR in the breast and ALNs (n = 11) developed metastases. ALN pCR was associated with a very low risk of developing metastases (1/18 patients). Persistent ALN disease was associated with a significant risk of developing metastases (10/25 patients).

**Figure 1:**
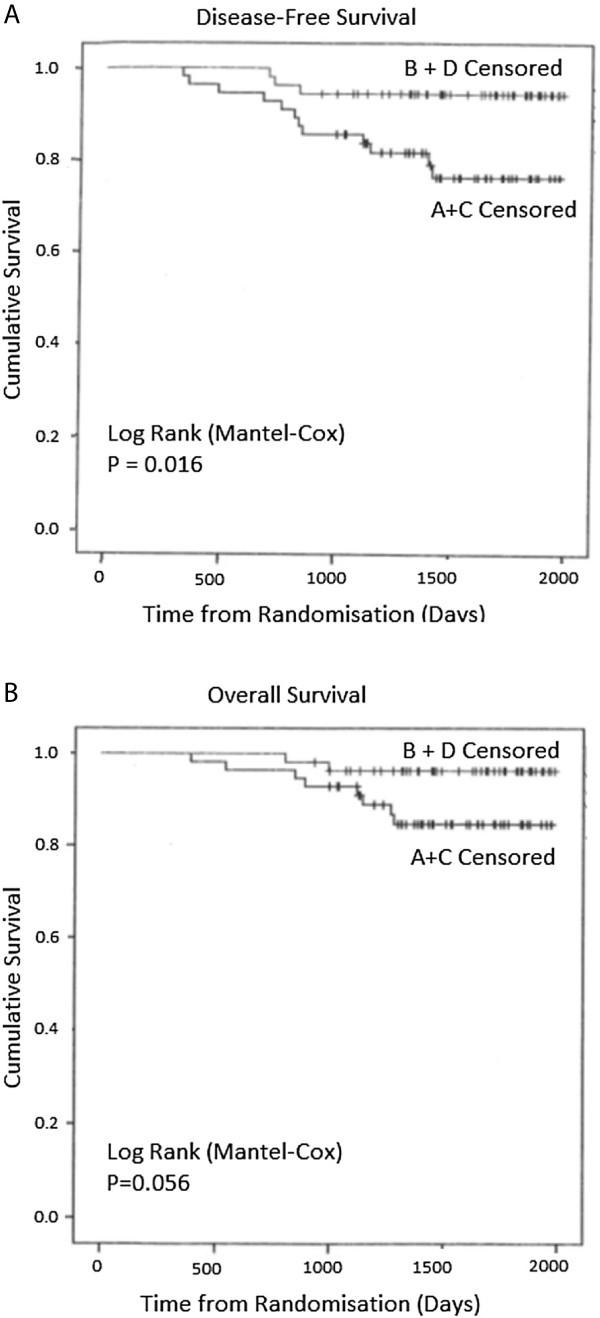
**Kaplan-Meier Survival curves for (A) DFS (** ***χ*** ^**2**^ **= 5.802, df1, p = 0.016) and (B) OS (** ***χ*** ^**2**^ **= 3.639, df1, p = 0.056) at a median follow-up period of 4 years and 3 months (Log Rank [Mantel-Cox]).**

The Kaplan-Meier DFS and OS (median follow-up of 51 months) are shown in Figure 1. Patients who received ACTX had a significantly increased DFS (Log Rank *χ* ^2^ = 5.802, p = 0.016). There were fewer events in OS, but there was a tendency for an improved short-term survival (Log Rank *χ* ^2^ = 3.639, p = 0.056).

## Discussion

PCR in the breast following NAC is a surrogate marker of improved survival (Kaufmann et al. 2012; von Minckwitz et al. 2012). In our study, pCR in the breast was defined as absence of invasive disease with or without ductal carcinoma *in situ* (DCIS). Various studies (5 to 10 year follow-up) have documented no change in DFS or OS in the presence of DCIS (Penault-Llorca et al. 2008; Mazouni et al. 2007; Marchio and Sapino 2011; Jones et al. 2006).

Cancer spread to ALNs results in poor survival and indicates systemic dissemination (Carter et al. 1989; Recht and Houlihan 1995). Residual disease in ALNs following NAC has a bad prognosis, even with a pCR in the breast (von Minckwitz et al. 2012; Marchio and Sapino 2011). PCR in the ALNs carries an excellent prognostis, even with residual invasive disease in the breast (von Minckwitz et al. 2012; Mazouni et al. 2007; Hennessy et al. 2005). The best DFS and OS occur when there is pCR in both the breast and axilla (von Minckwitz et al. 2012; Mazouni et al. 2007).

To achieve an improved pCR, various NAC combinations have been used, including capecitabine with anthracyclines and taxanes. The rationale for the use of capecitabine is the improved OS and time to disease progression with TX in advanced disease (O'Shaughnessy 2002; Gluck et al. 2013).

Two studies have reported an increased pCR in the breast with NAC-X combinations (Lee et al. 2008; Steger et al. 2010). Three others have failed to do so (von Minckwitz et al. 2010; Bear et al. 2012; Ohno et al. 2013). Our findings are in agreement with these latter results. Exclusion of DCIS reduced pCR responses to that documented by others using similar criteria (von Minckwitz et al. 2010).

Studies evaluating ALNs following NAC without capecitabine have documented a pCR of around 23% (Kuerer et al. 1999; Rouzier et al. 2002). Studies with NAC-X have shown no improvement (von Minckwitz et al. 2010; Bear et al. 2012). Our results are in agreement with these findings. Eleven patients with involved ALNs had a pCR in both nodes and breast; there was an almost 3-fold increase in those receiving ACTX.

Thirteen percent of the tumours were basal-like, comparable with other studies (Dent et al. 2007; Carey et al. 2007). These had the highest pCR (50%) as previously documented (Colleoni and Goldhirsch 2014; Colleoni et al. 2004). HER2 +ve tumours (luminal B, HER2 overexpressing) also elicited high levels of pCR, in agreement with published studies (Carey et al. 2007; Oh et al. 2006). The lowest pCR (17%) occurred in luminal A (ER +ve) tumours and is well established (Carey et al. 2007; Colleoni et al. 2004; Precht et al. 2010). Our study showed a significant correlation between pathological responses in the breast and tumour grade and HER2 status.

Two recent adjuvant studies did not demonstrate an improved 5-year DFS or OS with capecitabine (Ohno et al. 2013; Joensuu et al. 2012). However, patients with triple -ve cancers or 3 involved ALNs had an improved 5-year DFS and OS with capecitabine (Joensuu et al. 2012). Another study showed an increased OS but not DFS (O'Shaughnessy et al. 2009). A recent meta-analysis suggested an increased 5-year DFS and OS with adjuvant capecitabine (Jiang et al. 2012). In our study, capecitabine significantly improved the 4-year DFS (p = 0.016), but the OS just failed to reach significance (p = 0.056), probably due to the small number of deaths. Patients with basal-like and HER2 overexpressing tumours were at highest risk of metastases.

In metastatic disease the best results are obtained with TX (O'Shaughnessy 2002; Gluck et al. 2013). In our study, 84% completed 8 cycles of NAC. Of 55 patients receiving TX, 60% had a full dose. This is comparable to studies reporting no improved DFS with capecitabine (Ohno et al. 2013; Joensuu et al. 2012; O'Shaughnessy et al. 2009). Our planned dose of T was similar to that in two of the above trials, but 40-60% higher than in the third study. Our planned dose of X was 20-50% higher than in these three trials (Ohno et al. 2013; Joensuu et al. 2012; O'Shaughnessy et al. 2009).

The pattern and severity of the toxicity in our trial is comparable with other studies (Ohno et al. 2013; Joensuu et al. 2012). Severe hand-foot syndrome and nail changes were seen predominantly in patients receiving ACTX. Lenograstim substantially reduced the incidence of febrile neutropenia thereby contributing to reduced side-effects and improved QoL, as documented previously (Martin et al. 2006).

QoL did not differ significantly between groups on the primary outcome measures (FACT-B TOI) at any time point. The other measures of QoL in the various groups were comparable. The improved 4-year DFS did not occur at the expense of QoL.

Tumour stage at trial entry was positively correlated with HADS anxiety and depression and negatively with MRS happiness, clear headedness and relaxation, highlighting that women with LLABCs were distressed. This may have been due to their disease stage and proposed treatment, or biopsychological effects by the tumour (Walker et al. 2005). At baseline, depression correlated positively and mood (happiness, clear headedness) negatively, with clinical response to NAC; the more distressed the patient, the poorer the clinical response. Three weeks after completion of NAC, pathological response correlated positively with clear headedness and confidence. We have previously reported these relationships in women with LLABC undergoing NAC (different combination) and shown that distress was an independent prognostic factor for clinical and pathological responses (Walker et al. 1999).

Pathological response to NAC also correlated positively with tumour grade, and HER2 status.

Our study suggests a distinct benefit with NAC-X, with the doses of TX used in our trial, in patients with LLABCs. Further NAC studies with follow-up are needed to establish optimal TX delivery in patients at high risk to improve DFS and OS.

## Abbreviations

A: Doxorubicin
ALN: Axillary lymph node
ANCOVA: Analysis of co-variance
ANOVA: Analysis of variance
C: Cyclophosphamide
DCIS: Ductal carcinoma in situ
DFS: Disease-free survival
ER: Oestrogen receptor
FACT-B: Functional assessment of cancer therapy (Breast) with taxane (T) modules
HADS: Hospital anxiety and depression scale
HER2: Human epidermal growth factor receptor 2
LLABCs: Large and locally advanced breast cancers
MMG: Mammogram
MRM: Magnetic resonance mammogram
MRS: Mood rating scale
NAC: Neoadjuvant chemotherapy
OS: Overall survival
pCR: Pathological complete response
PR: Progesterone receptor
QoL: Quality of life
T: Docetaxel
TOI: Trial outcome index
USS: Ultrasound scan
WHO: World health organisation
X: Capecitabine.
